# Phase I randomized, observer-blinded, placebo-controlled study of a SARS-CoV-2 mRNA vaccine PTX-COVID19-B

**DOI:** 10.1038/s41598-023-35662-y

**Published:** 2023-05-26

**Authors:** Natalia Martin-Orozco, Noah Vale, Alan Mihic, Talya Amor, Lawrence Reiter, Yuko Arita, Reuben Samson, Queenie Hu, Anne-Claude Gingras, Bradley Thomas Sorenson, Eric Gates Marcusson, Piyush Patel

**Affiliations:** 1 Providence Therapeutics Holdings, 8832 Blackfoot Trail SE, Ste 120, Calgary, AB T2J 3J1 Canada; 2 Centricity Research, 2291 Kipling Avenue, Unit 117B, Toronto, ON M9W 4L6 Canada; 3 Department of Molecular Genetics, University of Toronto, 1 King’s College Cir, Rm 4396, Toronto, ON M5S 1A8 Canada; 4 Lunenfeld-Tanenbaum Research Institute at Mount Sinai Hospital, Sinai Health System, 600 University Ave, Toronto, ON M5G 1X5 Canada

**Keywords:** RNA vaccines, SARS-CoV-2

## Abstract

Access to vaccines against SARS-CoV-2 virus was limited in poor countries during the COVID-19 pandemic. Therefore, a low-cost mRNA vaccine, PTX-COVID19-B, was produced and evaluated in a Phase 1 trial. PTX-COVID19-B encodes Spike protein D614G variant without the proline-proline (986–987) mutation present in other COVID-19 vaccines. The aim of the study was to evaluate safety, tolerability, and immunogenicity of PTX-COVID19-B vaccine in healthy seronegative adults 18–64 years old. The trial design was observer-blinded, randomized, placebo-controlled, and tested ascending doses of 16-µg, 40-µg, or 100-µg in a total of 60 subjects who received two intramuscular doses, 4 weeks apart. Participants were monitored for solicited and unsolicited adverse events after vaccination and were provided with a Diary Card and thermometer to report any reactogenicity during the trial. Blood samples were collected on baseline, days 8, 28, 42, 90, and 180 for serum analysis of total IgG anti-receptor binding domain (RBD)/Spike titers by ELISA, and neutralizing antibody titers by pseudovirus assay. Titers in BAU/mL were reported as geometric mean and 95% CI per cohort. After vaccination, few solicited adverse events were observed and were mild to moderate and self-resolved within 48 h. The most common solicited local and systemic adverse event was pain at the injection site, and headache, respectively. Seroconversion was observed in all vaccinated participants, who showed high antibody titers against RBD, Spike, and neutralizing activity against the Wuhan strain. Neutralizing antibody titers were also detected against Alpha, Beta, and Delta variants of concerns in a dose dependent manner. All tested doses of PTX-COVID19-B were safe, well-tolerated, and provided a strong immunogenicity response. The 40-µg dose showed fewer adverse reactions than the 100-µg dose, and therefore was selected for a Phase 2 trial, which is currently ongoing.

Clinical Trial Registration number: NCT04765436 (21/02/2021). (https://clinicaltrials.gov/ct2/show/NCT04765436).

## Introduction

The coronavirus (severe acute respiratory syndrome coronavirus 2 [SARS-CoV-2]) worldwide pandemic that started in 2019 continues to affect multiple countries around the world where access to vaccines is limited^1^.

To provide an alternative prophylactic vaccine against SARS-CoV-2, Providence Therapeutics Holdings, Inc. (PT) developed an mRNA vaccine, PTX-COVID19-B, composed of a lipid nanoparticle containing modified mRNA that encodes for full-length Spike (S) protein with glycine in position 614 (G614). By March of 2020, the D614G variant of SARS-CoV-2 was the predominant strain worldwide and this substitution has prevailed in current, more transmissible variants of concern (VOCs)^2–4^. The recently described crystal structure of the full-length G614 S trimers indicates that G614 variant forms a more stable S trimer with one receptor binding domain (RBD) remaining in the up conformation longer than D614 in the pre-fusion state. The study posited that G614 S trimer stability could provide a superior immunogen for eliciting neutralizing antibody responses, largely targeting the RBD and N-terminal domain, which stay exposed longer than with the D614 S trimer^5^. Moreover, the study suggests that full-length G614 S protein vaccines could have a relative increase in potency because this change compensates for the lack of engineered stabilizing proline-proline (986–987 position) mutations present in other COVID-19 vaccines^5–8^. Preclinical studies showed PTX-COVID19-B was safe, highly immunogenic, and completely protected animals from SARS-CoV-2 infection^9^. Based on the preclinical results, PTX-COVID19-B was authorized by Health Canada to enter clinical trials in December of 2020.

Reported here are findings of the Phase 1 study to evaluate the safety, tolerability, and immunogenicity of PTX-COVID19-B vaccine in healthy seronegative adults aged 18–64. Our previous mouse studies indicated that immunizations with 10–20 μg of PTX-COVID19-B did not show any safety signal and could induce strong immune responses, therefore 16 μg was selected as the starting low dose for the Phase 1 trial. Mid and high doses were selected by increasing two and a half times the previous dose resulting in 40 μg and 100 μg respectively. The study began in January 2021 and enrollment was completed in April 2021. The safety database was locked, and analysis was performed on data up to day 42 after the first dose in May 2021. Immunological data is reported up to week 26 (day 180).

## Results

### Vaccine characteristics

PTX-COVID19-B vaccine is an off-white, sterile, preservative-free, frozen suspension for IM injection. It was produced as a multidose vial containing 2 mL of 0.2 mg/mL and supplied as a frozen suspension. Preparation of the vaccine for 16-µg, and 40-µg, dose was done by diluting the vaccine with saline to obtain 0.5 mL injection volume. No dilution was required for the 100-µg dose. Stability of PTX-COVID19-B was performed on vials stored at − 80 °C, − 20 °C for one year and the results indicate that at both temperatures the release specification stays constant. Stability for the vaccine stored at refrigerated conditions of 2–8 °C showed no changes in any of the release parameters for up to 3 months. Further stability studies are being conducted for more advanced stages of clinical development.

### Study demographics

**Figure 1:**
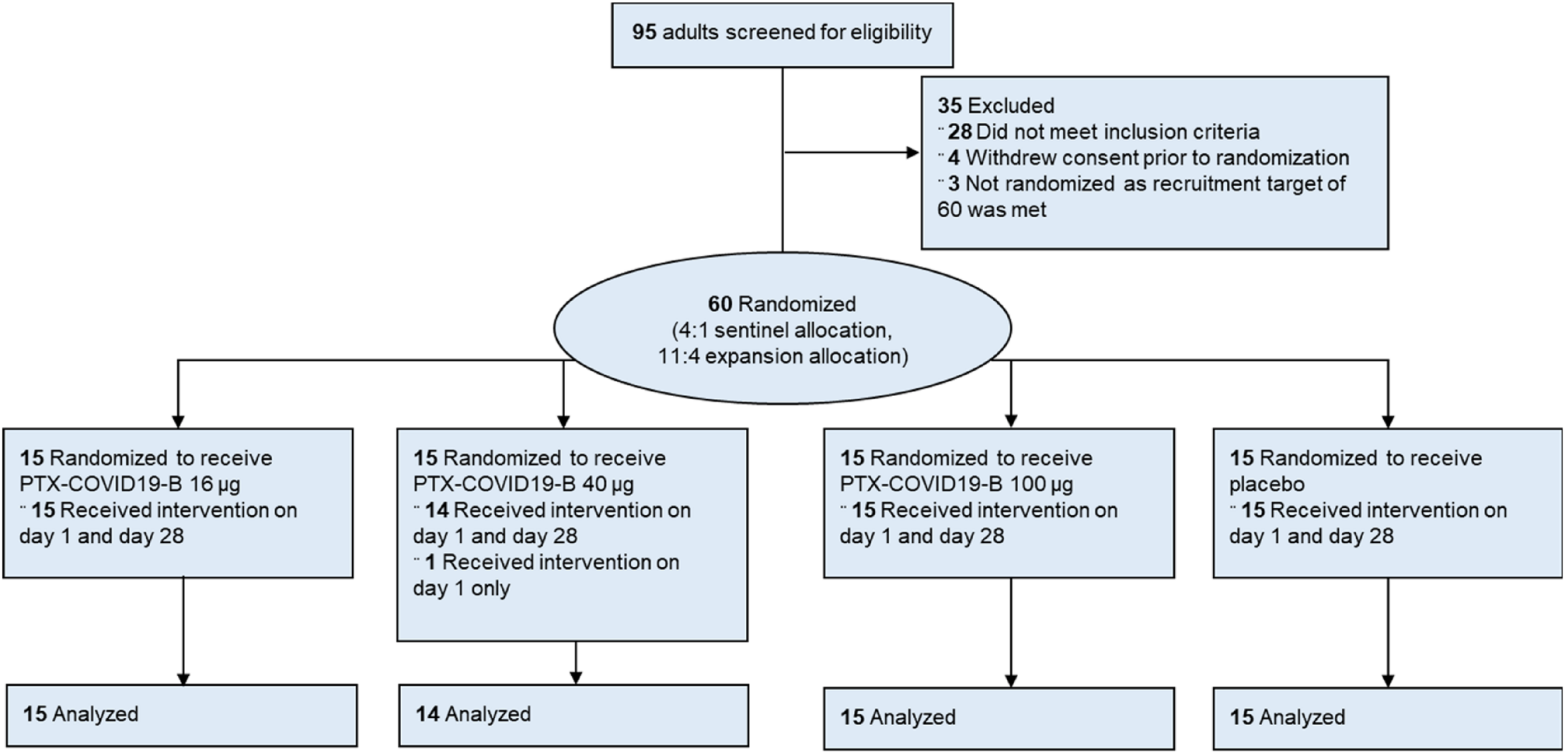
Study flow chart.

Sixty healthy adults were randomized into three groups comprising 20 participants each (Fig. 1). In each group, 15 participants received the PTX-COVID19-B vaccine and 5 received placebo. All 60 study participants received their first dose of vaccine on day 1 and all but 1 participant received a second dose on day 28. This participant, in the 40-µg dose group, withdrew from the study due to a non-study related consideration and was excluded from protocol population but still included in mITT population (modified intention to treat) final analysis.

The study included 30 (50%) female and 30 (50%) male participants. The mean age was 34.9 ± 12.95 years. Key demographic characteristics of the participants at enrollment are presented in Table 1.

**Table 1:** Demographic characteristics of the participants in the Phase 1 PTX-COVID19-B trial at enrollment.

Characteristic	PTX-COVID19-B 16 μg (N = 15)	PTX-COVID19-B 40 μg (N = 15)	PTX-COVID19-B 100 μg (N = 15)	Placebo (Pooled) (N = 15)	Overall (N = 60)
Sex—No. (%)					
Male	7 (47)	8 (53)	7 (47)	8 (53)	30 (50)
Female	8 (53)	7 (47)	8 (53)	7 (47)	30 (50)
Age—yr	38.3 ± 13.1	36.1 ± 15.5	32.5 ± 12.0	32.7 ± 11.1	34.9 ± 13.0
Race or ethnic group—No. (%)^a^					
American Indian or Alaskan Native	0	0	0	0	0
Asian	1 (7)	1 (7)	4 (27)	2 (13)	8 (13)
Black or African American	0	0	0	0	0
Native Hawaiian/Other Pacific Islander	0	0	0	0	0
White	13 (87)	13 (87)	11 (73)	13 (87)	50 (83)
Other	1 (7)	1 (7)	0	0	2 (3)
Hispanic or Latino	0	3 (20)	2 (13)	2 (13)	7 (12)
Body-mass index, kg/m^2b^	23.8 ± 3.34	25.3 ± 3.80	23.8 ± 3.11	23.9 ± 2.88	24.2 ± 3.28

Plus–minus values are means ± standard deviation.

^a^Race or ethnic group was reported by the participant.

^b^Body-mass index is the weight in kilograms divided by the square of the height in meters. This calculation was based on the weight and height measured at the time of screening.

### Safety

No serious adverse events, events leading to study discontinuation, events of special interest, new onset chronic disease, or potential immune-mediated medical conditions were reported.

**Figure 2:**
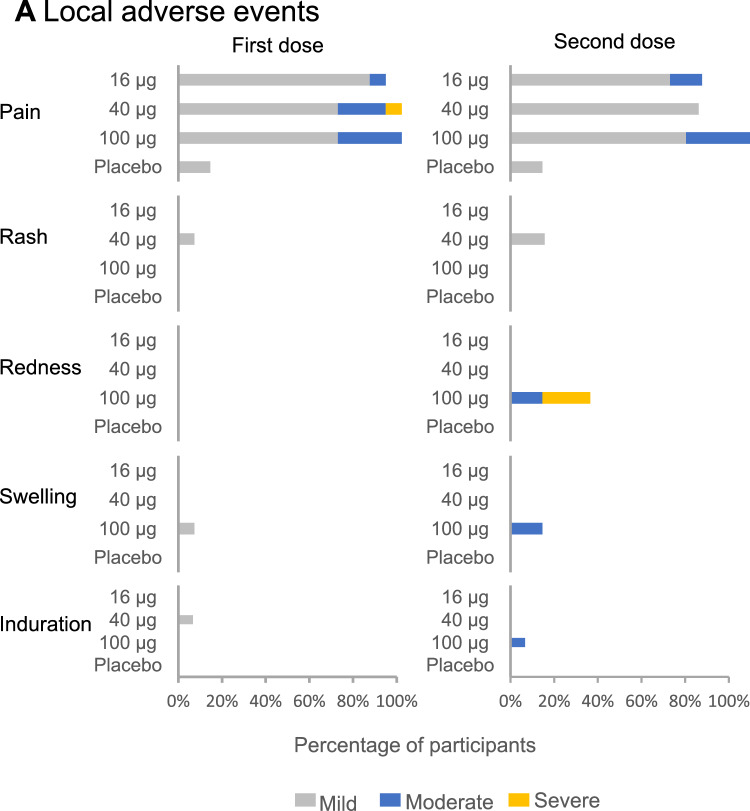

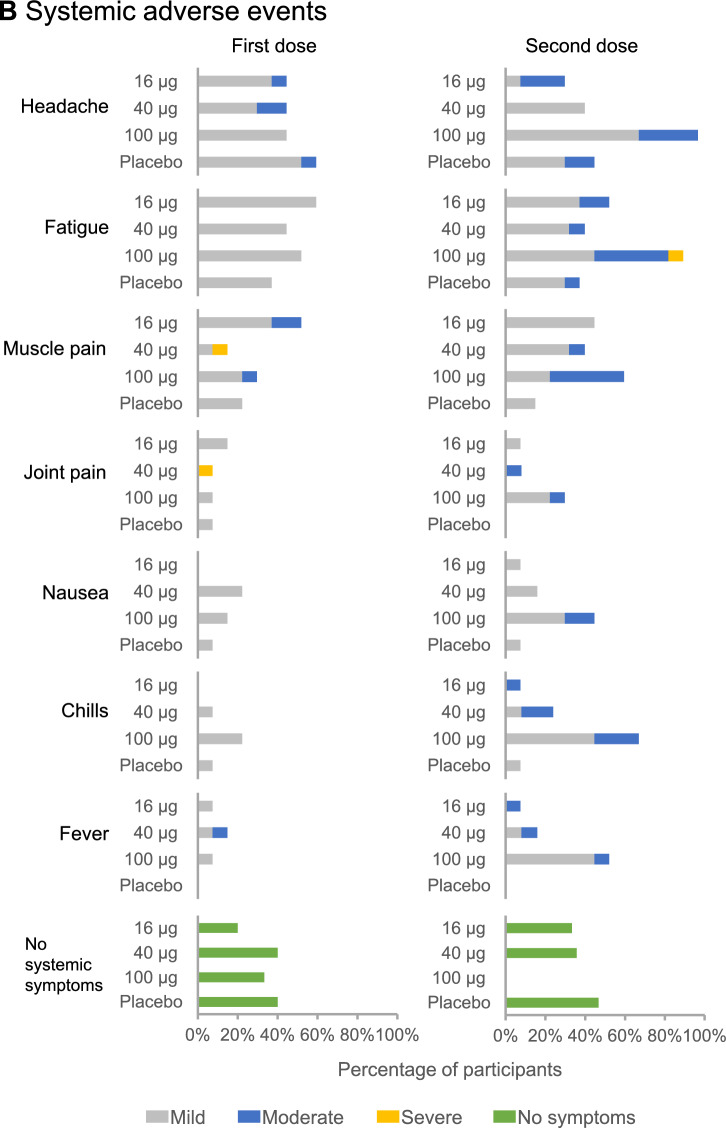
Summary of solicited systemic and local reactions. The plot represents the percentage of participants in each vaccine group (Cohort 1, 2, 3, and placebo) with local (Panel A) adverse events and solicited systemic (Panel B) adverse events through the third day post each vaccination according to FDA’s Toxicity Grading Scale (Grade 1, mild; Grade 2, moderate; Grade 3, severe; and Grade 4, potentially life threatening). There were no Grade 4 events. Participants who reported 0 events make up the remainder of the 100% calculation (not displayed). Participants that did not show any systemic symptom after vaccination are represented in the last row of panel B.

After the first dose, solicited systemic and local reactions were absent or mild in most participants. Two participants (14.3%) in Cohort 2 (40-μg dose) experienced severe solicited systemic reactions (muscle pain and joint pain) and 1 participant (6.7%) in Cohort 2 experienced a severe solicited local injection site reaction (pain) (Fig. 2). Participants that did not report any symptoms were 20% in Cohort 1, 40% in Cohorts 2 and 33% in Cohort 3.

After the second dose, solicited systemic and local reactions were absent or mild in most participants. One participant (6.7%) in Cohort 3 (100-μg dose) experienced a severe solicited systemic reaction (fatigue) and 3 participants (20.0%) in Cohort 3 experienced severe solicited local reactions (redness).

The incidence of solicited local and systemic reactions was highest at the 100-μg dose level, particularly after the second dose. Overall, the most commonly reported solicited local event was pain at the injection site and systemic adverse events were headache, fatigue, and muscle pain (Fig. 2). About 30% of the participants did not show any symptom after the second dose only in the Cohort 1 and Cohort 2.

The most frequently reported (> 5.0% across all cohorts) unsolicited treatment-emergent adverse events, defined as adverse events which occurred or worsened after the start of study vaccination, from day 1 to day 42, in the PTX-COVID19-B groups were injection site reaction (33.3% of participants in Cohort 3), diarrhoea (13.3% and 6.7% of participants in Cohorts 2 and 3, respectively), rhinorrhea (13.3% of participants in Cohort 1), and rash (13.3% of participants in Cohort 2). There were no reports of Grade 4 adverse event after any dose across groups. Most adverse events were mild in severity and unrelated to the study drug (except injection site reaction) (Fig. 2).

Four participants had medically attended adverse events, none of which were considered related to the study drug except severe injection site reactions in two participants in Cohort 2.

There have been no cases of COVID-19 reported up to week 26.

### Immunogenicity

#### Antibody response

**Figure 3:**
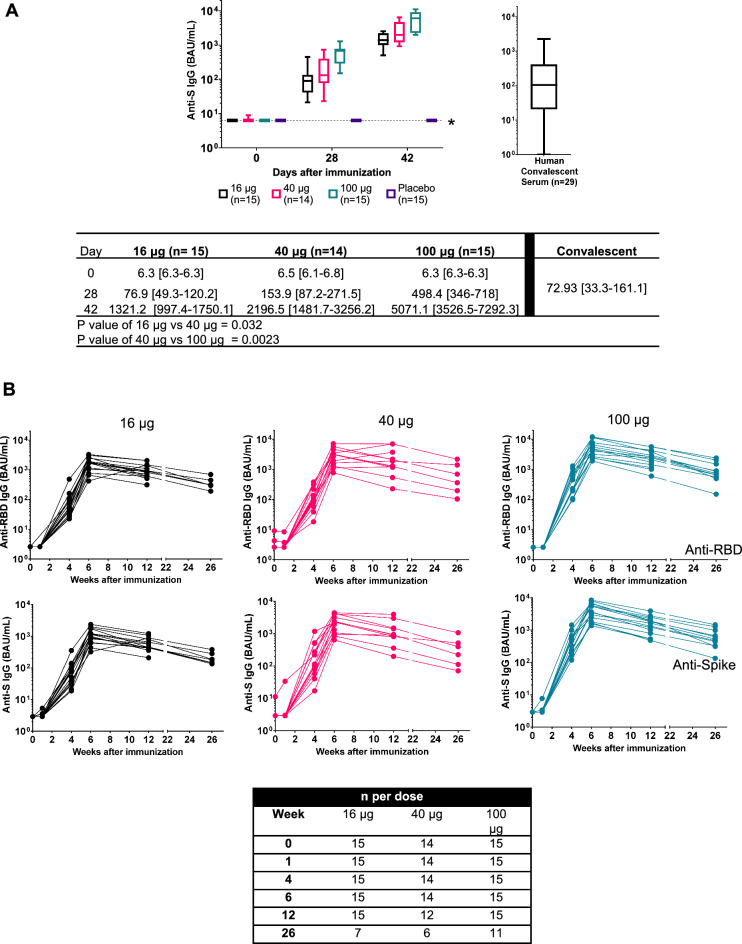
PTX-COVID19-B induction of anti-S and anti-RBD antibodies. Anti-S IgG antibody levels measured by ELISA are shown in Panel A. Anti-S antibody levels in sera from 29 subjects infected with COVID-19 were also determined by ELISA. The geometric mean and 95% confidence intervals are presented (*P* < 0.0001). Two tailed T test analysis was performed for titers differences on day 42 between 16 and 40 μg dose and 40 μg dose 100 μg and *P* values are indicated in the table. Anti-RBD and anti-S IgG antibody levels measured by MSD assay up to week 26 after immunization with PTX-COVID19-B are shown in Panel B. The number of subjects whose sera were tested at each timepoint are indicated in the table.

Eight days after immunization with PTX-COVID19-B there were no detectable antibodies against Spike or RBD either determined by enzyme-linked immunosorbent assay (ELISA) or Mesoscale Discovery (MSD) platform. By day 28 after the first immunization anti-S and anti-RBD immunoglobulin G (IgG) antibodies were detected across the three cohorts indicating seroconversion of all participants (Fig. 3) These antibodies were further significantly increased two weeks after the second dose of PTX-COVID19-B (day 42), in all three cohorts (*p* < 0.0001). Antibody levels induced by the vaccine was dose dependent and there was a significant difference between 16 μg dose and 40 μg (*p* = 0.032) and between 40 µg and 100 μg (*p* = 0.0023) serum samples by day 42. The antibody levels were monitored up to week 26 after the first vaccination (Fig. 3B) to evaluate duration of the immune response. Although the antibody levels decreased slightly by week 12 (day 84) and modestly by week 26 (day 180), they stayed above the levels observed after the first immunization (day 28). The week 26 antibody levels were also higher than COVID-19 convalescence serum except the low dose cohort (16-µg of dose, Cohort 1) (Fig. 3 and Supplementary Fig. S1). Both peak antibody level and week 26 antibody level were dose dependent.

The two assays for determination of anti-Spike antibodies, ELISA, and MSD, showed a strong positive correlation (correlation, 0.96 [95% confidence interval [CI] 0.95–0.98], *p* < 0.0001) (Supplementary Fig. S2).

#### COVID-19 neutralization antibody levels pre- and post-vaccination

**Figure 4:**
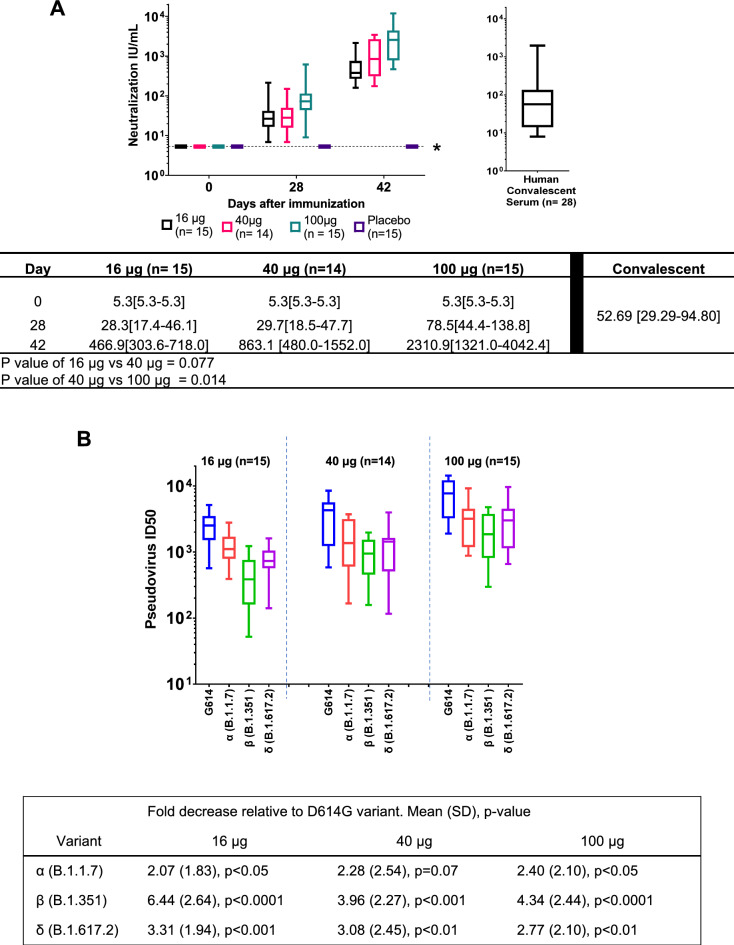
PTX-COVID19-B vaccine induction of neutralizing antibodies against G614 SARS-CoV-2 and VOCs. Levels of neutralization (IU/mL) of sera from PTX-COVID19-B subjects from day 42 against G614 are shown in Panel A. Levels of neutralization from sera from 28 subjects infected with COVID-19 were also determined. Two tailed t test analysis was performed for titers differences on day 42 between 16 and 40 μg dose and 40 μg dose 100 μg and *P* values are indicated in the table. Levels of neutralization (ID50) of sera from PTX-COVID19-B subjects from day 42 against VOCs Alpha, Beta, and Delta are shown in Panel B. Mean (SD) fold decrease relative to D614G variant are shown in the table, where values were calculated by dividing the average ID50 value of G614G by ID50 concentration of other variants indicated.

Neutralization activity was measured with a pseudovirus neutralization assay (pseudotype virus neutralization activity [pVNA] expressed in IU/mL as the assay was calibrated with the National Institute for Biological Standards and Control [NIBSC] standard) and was not detected in the participants before vaccination. After the first vaccination by day 28, neutralization activity was present in all participants sera of the three cohorts (Fig. 4A). Moreover, after the second vaccination pVNA levels increased 16-fold for the low dose, and 30-fold for the mid and high dose. The mean neutralizing antibody titers in PTX-COVID19-B vaccinated groups were at least eightfold higher at all doses compared to control serum samples from convalescent patients, indicated as human convalescent serum or HCS (Fig. 4A). The anti-S and anti-RBD antibody levels correlated with the neutralization units in all three assays (Supplementary Fig. S3). Also, the determination of angiotensin-converting enzyme 2 (ACE2):S blocking antibodies by MSD assay correlates with the neutralization units (Supplementary Fig. S4). Neutralization titers trended in a dose dependent manner, however statistical significance was not reached between Cohort 1 (16 µg dose) and Cohort 2 (40 µg dose) participants (*p* = 0.077), but it was reached between Cohort 2 and Cohort 3 (100 µg dose) (*p* = 0.014) (Fig. 4A).

Levels of neutralization against wild-type D614G and the Alpha (B.1.1.7), Beta (1.351), and Delta (B.1.617.2) VOCs were evaluated at day 42 for all three cohorts (Fig. 4B) using a pseudovirus neutralization assay. However, since there is no serum reference standard to calculate neutralizing Ab levels in international units per milli-liter available for VOCs, the results are expressed as ID50 (Inhibitory dilution 50%). All participants vaccinated with PTX-COVID-19-B produced neutralizing antibodies against the VOCs tested. As the vaccine dose increased, the ID50 against the VOCs also increased.

The magnitude of neutralizing antibody determined by the lentivirus assay and the recombinant Vesicular Stomatitis Virus (rVSV) neutralization assays correlated well (correlation: 0.87 [95% CI 0.80–0.91] *p* < 0.0001) for all samples from the three dose levels at day 28 and day 42 (Supplementary Fig. S5). The results indicate comparability of both assays for the interpretation of neutralization activity.

## Discussion

Vaccination with PTX-COVID19-B in 45 subjects of ages between 18- and 64-year-old did not result in any overt safety signal. Local reactions were mild after the first vaccine dose in general. Moderate to severe local pain was observed in a small number of participants (Cohorts 1 6%, and 26.7% for Cohorts 2 and 3). Similarly, systemic reactions were mild for most of the participants. Moderate headaches were observed in 6.7% of Cohort 1 and 13.3% of Cohort 2 participants. Moderate muscle pain was reported in 6.7% of Cohort 3 and 13.3% of Cohort 1 and severe pain was reported in 6.7% of Cohort 2.

After the second dose moderate pain at the injection site was similar to the first vaccine injection (13%, 0% and 26.7% for Cohorts 1, 2 and 3, respectively). However, moderate, and severe redness was observed in participants of Cohort 3 (33%). Also, moderate head aches and fatigue increase in Cohort 3–26% and 33% respectively whereas Cohort 2 showed systemic events only in 7.1% of the participants. These post second dose AE results let us to conclude that 16 μg and 40 μg would be preferred doses for vaccine development. Since the study is small, safety needs to be confirmed in follow up clinical trials with a larger number of participants^7,8,12^.

PTX-COVID19-B induced antibody titers against RBD and Spike remain high and above the values of COVID-19 convalescence serum over a period of 6 months (day 180) after initial vaccination, especially in the 40 μg and 100 μg dose cohorts, indicating a durable systemic antibody response.

Neutralizing antibody was observed in 100% of the vaccinated participants after the first immunization at day 28 regardless of the dose level. We speculate that this early induction of neutralizing antibody may be beneficial for vaccinees since it may provide early protection against SARS-CoV-2.

The neutralization serum activity post second immunization, determined using the Pseudovirus assay, was lower for the VOCs compared to the G614 strain. Fold titer reduction against Alpha was 2, against Delta was 3 in all three dose cohorts and against Beta was 4 in the 40 μg and 100 μg cohort. Some studies report similar findings with individuals vaccinated with approved mRNA vaccines, where the fold reduction against Alpha is 2, against Delta is 3–4 and more than 10 for Beta variants^10,11^. These results suggest that vaccination with PTX-COVID19-B induced antibody response that could cross protect against VOC, Alpha, Beta and Delta.

Studies proposing predictive models of COVID19 vaccine efficacy using data from approved vaccines, indicate that neutralizing antibody titers above twofold the value of HCS are predictive of over 80% efficacy protection against SARS-CoV2 ancestral strain infection^15^. This small phase 1 trial showed that PTX-COVID19-B induced neutralization titers, that are more than 8, 16 and 44 fold higher than convalescent sera at 16 μg, 40 μg and 100 μg respectively (*P* < 0.001). These results suggest that PTX-COVID19-B could potentially be an efficacious vaccine candidate against Wuhan derived strains. Moreover, the strong immunogenicity of PTX-COVID19-B indicates that the full-length expression of Spike without the proline-proline stabilization, can produce a good Spike target for vaccine development. All these potential advantages of PTX-COVID19-B candidate vaccine need to be further evaluated in follow-up clinical trials.

The 40-µg dose was selected to progress into a Phase 2 clinical trial, because it had the right balance between the immune response and satisfactory reactogenicity profile.

Critical limitations of this trial include a small study population and limited ethnic diversity as compared with the general population. Additionally, as a Phase 1 safety trial, the study population did not include those at increased risk of severe illness from COVID-19, including older adults and immunocompromised individuals that may mount a weaker immune response to vaccination.

Although several COVID-19 vaccines have been approved globally (most under emergency or interim use), issues remain with supply and delivery of vaccines to combat emerging variants. Additional safe, effective, and easily deployable SARS-CoV-2 vaccines are needed to meet the challenge for global immunization required to end the pandemic. Based on the reported results, PTX-COVID19-B vaccine is a promising candidate that warrants testing in the next phase of trials. A Phase 2 comparator trial of PTX-COVID19-B and Comirnaty in 525 healthy adults with the 40-µg dose has been performed and data is being analyzed for a future report. T cell responses were evaluated in a significant number of participants of the Phase 2 trial to complete the immune response profile of the vaccine.

The sera from subjects vaccinated with PTX-COVID19-B did not cross neutralize Omicron Spike expressing pseudovirus (data not shown). These results were expected since Omicron Spike from the new VOC differ in more than 30 amino acids from D614G Spike. Based on the performance of the mRNA and LNP of PTX-COVID19-B, future booster vaccine development is planned with updated Spike versions of circulating strains.

## Methods

### Trial design

This observer-blinded, randomized, placebo-controlled, ascending dose Phase 1 study is being conducted at one site in Canada (Manna Research, Toronto, Ontario). Eligible participants included healthy men and nonpregnant women between 18 and 64 years of age, with a body mass index of 18–30 kg/m^2^ at screening. All participants were seronegative to SARS-CoV-2 and reverse transcription-polymerase chain reaction (RT-PCR)-negative without evidence of recent exposure to SARS-CoV-2 or viral respiratory diseases.

Two doses of 16-µg (Cohort 1), 40-µg (Cohort 2), and 100-µg (Cohort 3) PTX-COVID19-B were compared to placebo with 20 participants in each cohort. Vaccine or placebo were administered on days 1 and 28. The sentinel group had five participants in each of the dose cohorts, who were randomized in a 4:1 ratio (vaccine: placebo). After review of the safety data of the sentinel group, the trial was followed by a cohort expansion of 15 participants randomized in a 11:4 ratio (vaccine:placebo). Randomization codes were generated using a computer program and provided as a paper listing of vaccine or placebo assignment by an unblinded statistician. The pharmacist and staff members that prepared and administered the doses based on the paper randomization were unblinded; all others involved in the conduct of the study (Principal Investigator, site coordinator, site staff) and the participants were blinded.

PT was the sponsor of the study, which was conducted under a protocol approved by Advarra. This study was conducted in accordance with the provisions of the Declaration of Helsinki, and in accordance with the International Council for Harmonisation of Technical Requirements for Pharmaceuticals for Human Use E6 Guidelines on Good Clinical Practice (GCP). All participants provided written informed consent prior to enrollment. Full text of the trial protocol, including detailed statistical analysis plan are provided in the Supplementary Information.

### Trial vaccine and placebo

PTX-COVID19-B mRNA vaccine, manufactured by PT (lot number: PTX-CB-003), was supplied as a sterile solution (0.2 mg/mL). The mRNA encoding for 1273 amino acid of SARS-COV-2 whole length S protein was formulated in lipid nanoparticles as the final drug product. The placebo was injectable saline solution (sodium chloride 0.9%). Vaccine doses were diluted with injectable saline to reach the 16-µg and 40-µg doses. The 100-µg doses did not require dilution. Vaccine and placebo were administered intramuscularly into the upper arm deltoid muscle of the non-dominant side in a 0.5 mL dose with a 25G needle.

### Safety and reactogenicity assessments

Participants were monitored for solicited and unsolicited adverse events (see Supplementary Fig. S6 for the schedule of key assessments). The investigator assessed each event for seriousness, intensity, and relationship to the vaccine. Vital signs and site reactions were monitored for an hour after each vaccination. Participants maintained a Diary Card to track reactogenicity and other safety assessments for 7 days after each vaccination. Reactogenicity solicited events included systemic symptoms including headache, fatigue, muscle pain, joint pain, nausea, chills, and fever, and localized reactions including injection site pain, bruising, redness, and swelling. Participants were asked to record oral temperature with a thermometer if they felt any of the above symptoms and record it in the Diary Card.

Additionally, the overall safety was analyzed, including unsolicited adverse events, medically attended adverse events, new onset chronic disease, serious adverse events, adverse events of special interest, potential immune-mediated medical conditions, and findings from targeted physical examinations, vital signs assessments, and clinical safety laboratory testing. The intensity of a solicited adverse event was graded according to United States Food and Drug Administration (FDA) standards (see Supplementary Table S1)^17^. For the adverse events not covered by the FDA standard, the rating scale from the Division of AIDS (DAIDS) was used (see Supplementary Table S2)^18^. Standard laboratory and vital signs reference ranges were used and assessed for clinical significance by the Investigators.

Safety reviews were performed by the independent Safety Review Committee according to the committee charter.

### Immunogenicity assessments

#### Anti-S and anti-RBD MSD assay

The responses induced by PTX-COVID19-B on anti-COVID-19 S-protein IgG and anti-COVID-19 RBD IgG were analyzed from serum samples using a multiplex immunoassay sandwich-based method with an electrochemiluminescent (ECL) readout on the MSD platform. Diluted samples as well as calibrators and controls were added to MSD plates (multi-spot wells) coated with either the S or RBD SARS-CoV-2 antigens to capture human IgG antibodies present in the samples. Bound IgG antibodies were then detected using sulfo-TAG labeled anti-human IgG antibody. The human IgG antibody concentration was determined by interpolation of the ECL count on the calibration curve. The assay was validated and performed under GLP conditions at CIRION (Laval, Quebec; V-PLEX® SARS-CoV-2 Panel 2 Kit). Analysts knew timepoints but not dose levels or regimen of the samples. To convert the results from MSD arbitrary units (AU/mL) to the World Health Organization (WHO) International Standard in binding antibody units (BAU)/mL, a conversion factor of 0.0272 was applied for anti-RBD IgG concentrations and 0.00901 for anti-S IgG concentrations. Additionally, anti-S and anti-RBD IgG measurements for 21 control serum samples from convalescent patients with a documented positive SARS-CoV-2 RNA diagnosis (mentioned in the test as Human Convalescent Sera or HCS) were also performed (Supplementary Fig. S1). The HCS samples provided by the laboratory were obtained as a donation from volunteers. No demographic or time from infection were provided.

#### Anti-S ELISA

An indirect binding ELISA was also performed to determine if the elicited antibodies could bind the S protein. The SARS-CoV-2 pre-fusion S antigen was adsorbed onto a 96-well microplate and standard ELISA procedure was followed with anti-SARS-CoV-2 S IgG specific antibody (primary antibody) and anti-human IgG antibody (secondary antibody) conjugated to peroxidase. The absorbance of each well was measured using a spectrophotometer at a specific wavelength (450/620 nm). A standard on each tested plate was used to calculate the antibodies against SARS-CoV-2 S according to the unit assigned by the standard (ELU/mL). The assay was validated and performed under GLP conditions at Nexelis a Q^2^ Solutions Company (Laval, Quebec). To convert the results to the WHO International Standard in BAU/mL, a conversion factor of 1/7.9815 was applied. Additionally, anti-S IgG ELISA measurements for 28 control serum samples from convalescent patients with a documented positive SARS-CoV-2 RNA diagnosis (mentioned in the test as Human Convalescent Sera or HCS) were also performed. Demographics, time from infection or any personal data from HCS donors were not provided by the laboratory.

#### Pseudovirus neutralization VSV-based assay

To evaluate the neutralizing effect of the sera from participants treated with PTX-COVID19-B, a pseudotyped virus neutralization assay developed and validated at Nexelis, Canada to quantify the titer of neutralizing antibody against SARS-CoV-2^19^. The assay used rVSV particles containing modified S protein of SARS-CoV-2 (Wuhan strain) for which the last 19 amino acids of the cytoplasmic tail were removed. The pseudoparticles contained a luciferase reporter for quantification in relative luminescence units (RLU). The dilution of plasma required to achieve 50% neutralization (NT50) when compared to the pseudoparticle control was interpolated from a linear regression using the two dilutions flanking the 50% neutralization. To convert the NT50 titer to the WHO International Standard in IU/mL, a conversion factor of 1/1.872 was applied. Additionally, neutralizing antibodies for 28 control samples from Human Convalescent Sera (HCS) were assessed in the pseudovirus (rVSV) neutralization assay.

#### Pseudovirus neutralization lentivirus-based assay

To determine neutralization activity against SARS-CoV-2 Wuhan-Hu1 (D614G) and the VOCs, a pseudovirus neutralization assay was performed using a SARS-CoV-2 S protein pseudotyped lentivirus at the Lunenfeld-Tanenbaum Research Institute at Mount Sinai Hospital (Toronto, Ontario). Spike-pseudotyped lentiviral assays were performed as previously described with minor modifications^9,16^. The pseudotyped virus used in this assay is a lentivirus expressing the S protein of SARS-CoV-2 Wuhan-Hu1 (D614G), or the Alpha (B.1.1.7), Beta (B.1.351), or Delta (B.617.2) variants. Spike-pseudotyped lentivirus particles were generated and used at a virus dilution resulting in > 1000 RLU over control. For the neutralization assay, human sera were serially diluted and incubated with diluted pseudovirus at a 1:1 ratio for 1 h at 37 °C followed by transfer to plated HEK293T-ACE2/TMPRSS2 cells and incubated for 48 h at 37 °C and 5% CO_2_. Cells were then lysed, and Bright Glo luciferase reagent (Promega, Madison, WI) was added for 2 min prior to reading with a PerkinElmer Envision instrument (PerkinElmer, Waltham, MA). The ID50 were calculated in GraphPad Prism 9 using a nonlinear regression algorithm (log[inhibitor] versus normalized response—variable slope). The assay was performed in the same manner for all VOCs tested.

### ACE2:S neutralization assay

The neutralizing antibody response was analyzed at days 8, 28, and 42 using a S-ACE2:S blocking assay which has an ECL readout on the MSD platform. The assay measures antibodies that block the binding ofACE2 to the SARS-CoV-2 S protein and RBD antigens. Blocking antibodies bind to S protein in the plate and prevent the binding of ACE2 labeled with MSD-Sulfotag. A reference standard, included in the kit, was used to generate a standard curve. Neutralizing antibody concentrations in samples were calculated by backfitting the measured signals for samples to the standard curve. Correcting for dilution provided the final neutralization antibody concentration in undiluted samples. In this assay, 1 unit/mL corresponded to neutralizing activity of 1000 ng/mL of antibody that can bind to S protein.

### Statistical analysis

The statistical analysis was conducted using SAS^®^ version 9.04 (SAS Institute, Cary, NC). No formal sample size calculation was performed.

The database was locked and unblinded five weeks after the last participant received their last dose. The data presented here are up to 6 months (day 180) for anti-RBD and anti-S IgG antibody levels by MSD and up to day 42 for all other safety and immunogenicity analyses, two weeks after the last participant received their last dose.

Immunogenicity data were summarized by cohort and time point using descriptive statistics along with geometric mean and 95% CI. Results were analyzed using a mixed effects model with treatment as a fixed effect and participant as a random effect for each visit. Placebo participants were pooled for comparison. ANOVA with Tukey’s multiple comparison was performed (F [3, 55] = 26.90, degrees of freedom = 3). Analysis was performed at a 5% level of significance. The correlation coefficients were calculated using a two-tail Pearson correlation with 95% confidence.

The number and percentage of participants reporting any treatment-emergent adverse event or reactogenicity were summarized by cohort and tabulated by system organ class and preferred term (coded using MedDRA, WHO Global B3 format—MAR 2021).

## Supplementary Information

Supplementary Information.

## Data Availability

The datasets generated during and/or analysed during the current study are available from the corresponding author on reasonable request.
